# Supplementary material for: Peroxisome Proliferator-Activated Receptor Alpha Stimulation Preserves Renal Tight Junction Components in a Rat Model of Early-Stage Diabetic Nephropathy
Source: Int J Mol Sci. 2024 Dec 7;25(23):13152. doi: 10.3390/ijms252313152 (PMC11641852; doi:10.3390/ijms252313152)
Supplement: Supplementary file 1 [file ijms-25-13152-s001.zip › ijms-3313298-supplementary.pdf]

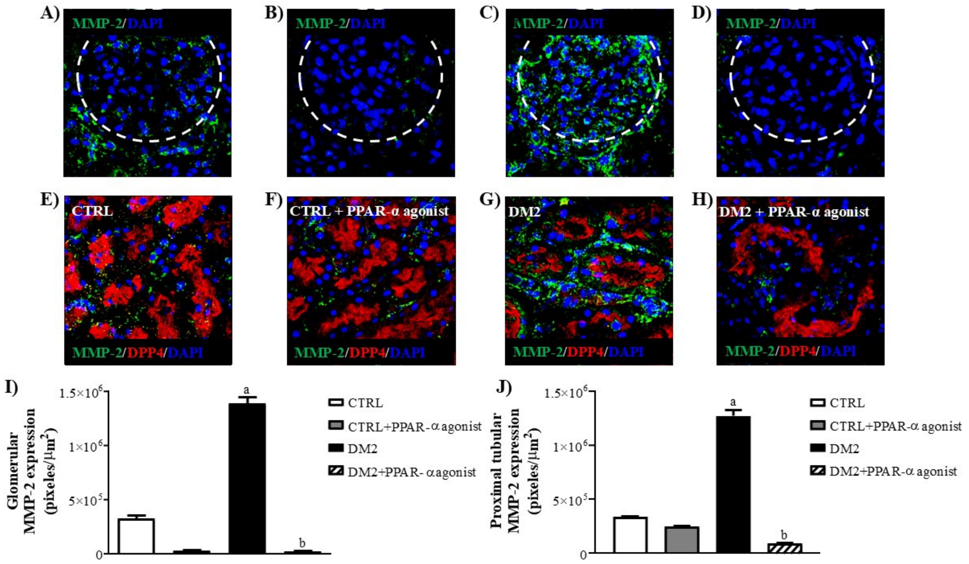

Supplementary Figure S1. MMP-2 expression in glomeruli and proximal tubules by immunofluorescence. Representative immunofluorescence images from kidney sections glomeruli (A-D) and proximal tubules (E-H) of four experimental groups showing MMP-2 in green, DPP4 in red as a proximal tubule marker, and DAPI as a nuclear marker A and E) CTRL, B and F) CTRL+PPAR-α agonist, C and G) DM2, and D and H) DM2+PPAR-α agonist. Bar = 50 µm. B-C. Quantification of the I) glomerular and J) proximal tubular MMP-2 expression, respectively. The bars represent the mean value ± SEM (n= 4 rats evaluated per group). a p<0.05 versus CTRL, and b p<0.05 versus DM2 from a one-way ANOVA followed by a post-hoc Tukey test

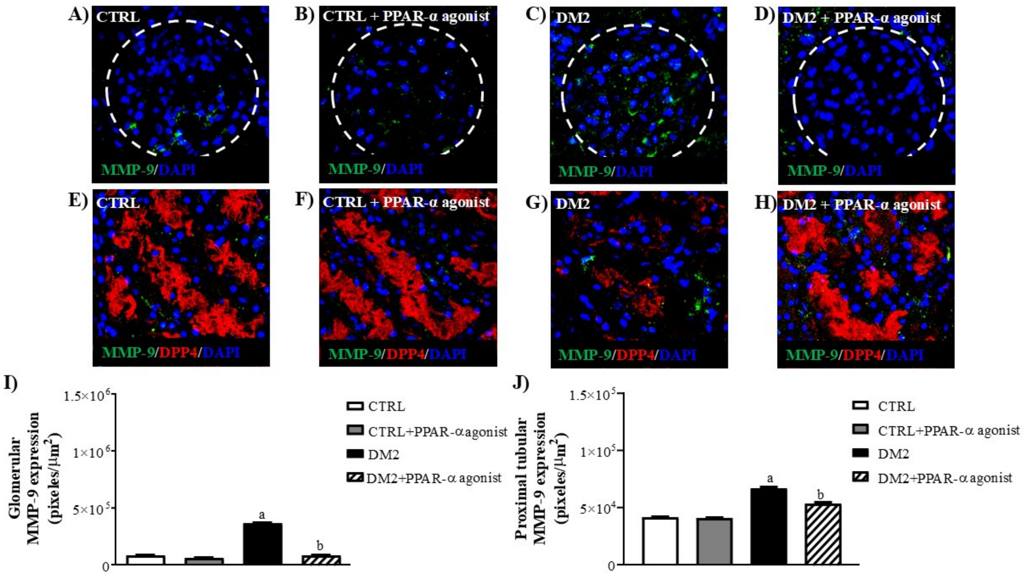

Supplementary Figure S2. MMP-9 expression in glomeruli and proximal tubules by immunofluorescence. A) Representative immunofluorescence images from kidney sections glomeruli (A-D) and proximal tubules (E-H) for the four experimental groups showing MMP-2 in green, DPP4 in red as a proximal tubule marker, and DAPI as a nuclear marker A and E) CTRL, B and F) CTRL+PPAR-α agonist, C and G) DM2, and D and H) DM2+PPAR-α agonist. Bar = 50 µm. Quantification of the I) glomerular and J) proximal tubular MMP-9 expression, respectively. The bars represent the mean value ± SEM (n= 4 rats evaluated per group). a p<0.05 versus CTRL, and b p<0.05 versus DM2 from a one-way ANOVA followed by a post-hoc Tukey test.

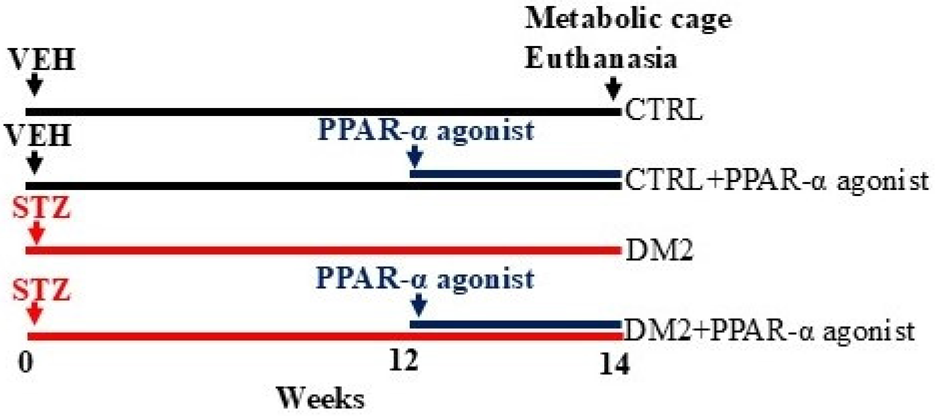

Supplementary Figure S3. Diagram of experimental design showing the main experimental groups and the subdivision at 12-week to start PPAR-α agonist treatment. Treatment was administered for two weeks and sacrificed at finished it.
